# Visual attraction of the European tarnished plant bug *Lygus rugulipennis* (Hemiptera: Miridae) to a water trap with LED light in chrysanthemum greenhouses and olfactory attraction to novel compounds in Y‐tube tests

**DOI:** 10.1002/ps.6881

**Published:** 2022-04-06

**Authors:** Robert WHM van Tol, Celia M Diaz Rodriguez, Antje de Bruin, Daowei Yang, Tanvi Taparia, Frans C Griepink

**Affiliations:** ^1^ Wageningen University and Research Wageningen The Netherlands; ^2^ Present address: BugResearch Consultancy Wageningen The Netherlands; ^3^ Present address: Entocare Biocontrol C.V. Wageningen The Netherlands; ^4^ Present address: Pherobank B.V. Wijk bij Duurstede The Netherlands

**Keywords:** LED light, pheromone, kairomone, plant odor, olfactometer, GC‐EAD, GC–MS

## Abstract

**BACKGROUND:**

The European tarnished plant bug *Lygus rugulipennis* Poppius (Hemiptera: Miridae) can cause several types of damage to crops grown in greenhouses and fields, including flower abortion in eggplant, stem and fruit damage in cucumbers, and splits in chrysanthemums. Studies suggest that both male and female *L*. *rugulipennis* may be more attracted to traps based on visual attraction than pheromone‐based trap. Therefore, the aim of this study was to evaluate the effectiveness of a water trap with LED lights and semiochemicals in trapping *L*. *rugulipennis* in the laboratory and greenhouse.

**RESULTS:**

The results showed that water traps equipped with white LED light caught 20– 30 times more bugs than did the sex pheromone‐based traps in greenhouse experiment. During the week of peak flight, the LED water trap caught a total of 29 males and females, whereas the sex pheromone caught only one male. Among the semiochemicals tested in a Y‐tube, both males and females were attracted to *ß*‐caryophyllene, but not in the presence of the sex pheromone, whereas both males and females were attracted to pentyl butyrate in the presence of the sex pheromone. The pheromone plus bean plant odor was attractive to the insects, suggesting an interaction between plant odor and pheromone.

**CONCLUSION:**

Overall, the findings of the study showed that the water trap with LED light could be an effective method for trapping *L*. *rugulipennis* in greenhouses. © 2022 The Authors. *Pest Management Science* published by John Wiley & Sons Ltd on behalf of Society of Chemical Industry.

## Graphical abstract

An attractive water trap with white LED light catches 20 to 30 times more female and male *Lygus rugulipennis* than the sex pheromone trap.

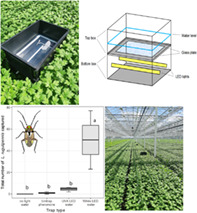

## 1 INTRODUCTION

The European tarnished plant bug, *Lygus rugulipennis* Poppius (Hemiptera: Miridae) is one of the most polyphagous pest insects in the world,^1^ affecting 437 plants from 57 families. Most host plants belong to the Brassicaceae, Asteraceae, and Fabaceae family. The most prominent damage is found in economically important crops such as chrysanthemum, eggplant, cucumber, peppers, and strawberry; however, this does not necessarily represent the order of prevalence of this bug. Studies are yet to clarify if *L*. *rugulipennis* has a preferred plant species. Approximately 9–10 *Lygus* species have been identified as crop pests in North America and Europe.^2^, ^3^ *Lygus* species inject salivary enzymes through piercing‐sucking mouthparts, mainly in meristematic tissues, such as apical buds and fruits, resulting in the death of tissue in and around the feeding site and malformation of the tissue. Presently, effective active ingredients against *Lygus* species available in the EU are limited, necessitating the development of sustainable and eco‐friendly alternative approaches for pest control.^4^

Presently, two control strategies have been adopted against *Lygus* species in greenhouse crop production systems, which includes the use of systemic insecticides^5^, ^6^, ^7^ to control the invasion of migrating adults into greenhouses, and the use of entomopathogenic fungi as part of integrated pest management (IPM) strategies.^6^, ^7^, ^8^ However, these methods are ineffective because of their slow mode of action, especially against greenhouse‐invading adults that cause immediate damages. Mass‐trapping with pheromones^8^ and more recently a ‘push‐pull’ strategy^4^ are promising novel techniques for pest control. Fountain *et al*.^9^ reported that mass trapping could be more effective than biocontrol (entomopathogenic fungi) in preventing insect damage, especially, immediate damage caused by adult bugs entering the greenhouse. Furthermore, a ‘push‐pull’ strategy, as demonstrated by Fountain *et al*.^4^ is promising for the control of settled and reproducing populations in the greenhouse. Additionally, nymphs of the bugs could also cause damage and cannot be trapped by flight, but by a combination of push–pull strategy and mass‐trapping of adults this may become a successful control strategy. Mass‐trapping of migrating bugs entering the greenhouse requires a more powerful attractive trap (visual and olfactory). Physical methods preventing migrating bugs from entering the greenhouse via vents are rarely used in the Netherlands, mainly because of high maintenance costs.

Studies have shown that the currently available sex pheromone‐based (contains an unknown (not published) composition of the compounds hexyl butyrate, (*E*)‐2‐hexenyl butyrate and (*E*)‐4‐oxo‐2‐hexenal) trap in the market is less effective and can only capture male *L. rugulipennis* in small numbers.^10^, ^11^ Presently, neither aggregation pheromones^12^, ^13^ nor kairomonal compounds^14^, ^15^, ^16^, ^17^ have been effective in trapping *Lygus* species.

In the absence of attractants, a highly effective visual trap may increase the trap catch of the bugs. Presently, a green Unitrap consisting of a bucket with a funnel entrance and green cross‐vanes on top is used to trap *L. rugulipennis* in combination with the sex pheromone.^9^, ^11^ However, these traps have low efficacy, with only 7.1% of bugs captured.^11^ This low trap efficacy is not surprising since *L. rugulipennis*, a dusk flying/migrating insect is similar to most other *Lygus* species,^18^ making visual orientation to sunlight reflecting material unlikely to be effective. Although a study on sun‐reflecting sticky colored plates showed that *L. rugulipennis* was more attracted to blue colours,^19^ literature on visual orientation is limited, indicating the importance of studying color preference in trapping bugs. Several *Lygus* spp. are dusk/night migrating species, and some variations in the time of flight during the night have been observed for different species. Šedivý and Honěk^18^ concluded that the migration flights of *L. rugulipennis* appears to be between 6 pm and 1 am. For other related species such as *L. hesperus*, peak flight was found between 3 pm and 7 pm.^20^ The main period of nocturnal flight activity of *L. rugulipennis* is from late June to October, and usually has two or three peaks.^18^ A study on the influence of polarized moonlight on attraction to light‐traps showed that both *L. rugulipennis* and *L. pratensis* were caught in light traps at full moon (high polarized light).^21^ Clearly *L. rugulipennis* is a dusk/night migrating species. Šedivý and Honěk^18^ reported that traps consisting of 250 W mercury vapor lamp (luminosity 1200 l m, approximately 25% UV light) shining on a white panel with a grid of electric wires in front to collect the insect after electrocution, was effective against *L. rugulipennis*. However, the role of UV or light wavelength on the attraction of *L. rugulipennis* is unknown. For instance, a study showed that *Nesidiocoris tenuis* (Reuter) is strongly attracted to UV‐A light at a wavelength of 365 to 385 nm.^22^ Furthermore, LED lights at specific wavelengths could be effective in capturing *L. rugulipennis*. However, studies are yet to examine the effect of LED lights and different wavelengths in capturing bugs.

Therefore, the aim of this study was to evaluate the effectiveness of water trap with LED lights and semiochemicals in trapping *L*. *rugulipennis* in the laboratory and greenhouse. To achieve this, the attraction of *L. rugulipennis* to different light wavelength was examined. Based on the wind tunnel results, the efficacy of the water trap with LED light was evaluated in two chrysanthemum greenhouses. Furthermore, the attraction of the bugs to minor compounds found in the extracts of the bugs that may function as aggregation pheromones, as well as compounds of host plants when damaged by *L. rugulipennis*, was examined. Additionally, the headspace of various plant and bug fractions were analyzed by GC–MS to identify unique compounds. The response of the bugs to these compounds and previously identified potential kairomones,^9^, ^12^, ^15^, ^16^ was examined in Y‐tube olfactometer experiments.

## 2 MATERIALS & METHODS

### 2.1 *Lygus rugulipennis* rearing and use in experiments

Cultures of *L. rugulipennis* were maintained on bean pods (*Phaseolus vulgaris* L.) at 20 °C (16 h/8 h light/dark photoperiod) in Entocare C.V. laboratory for use in combination with semiochemicals in Y‐tube experiments or collected from chamomile and used directly in Y‐tube experiments (unknown age). Males and females (Entocare culture) were separated shortly after 5th instar nymphs emerged into adults and kept on bean pods at 20 °C and 70% relative humidity (RH) until use. Five‐to seven‐day‐old adult male or female bugs were used for odor trapping by plants and for olfactory experiments with the Y‐tube. Additionally, the attraction of wild‐collected bugs from chamomile to plant and pheromone in the Y‐tube olfactometer was examined to determine possible variations in response. The visual wind tunnel experiments were performed using 1–3 days old adults cultured on bean pods (T = 20 °C, RH = 70%). The response of female and male bugs to different LED light wavelength sources were examined using the wind tunnel experiment. Adults were starved for 24 h prior to the experiments.

### 2.2 Wind tunnel visual experiments

#### 2.2.1 Wind tunnel settings

**Figure 1:**
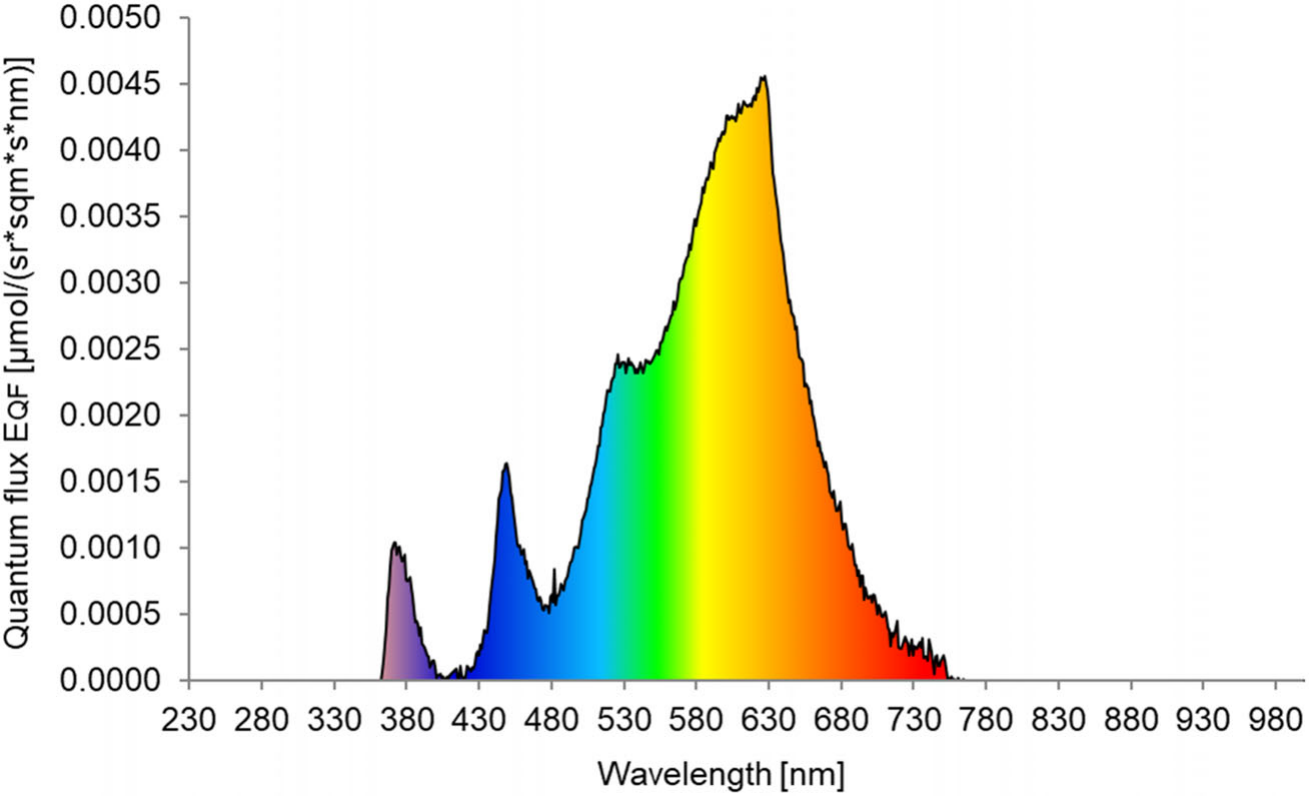
Light reflection of the ceiling in a wind tunnel measured as reflection on a spectralon plate (μmol photons per steradian m^−2^ s^−1^ nm^−1^). Total measured reflection is 0.64 μmol photons per steradian m^−2^ s^−1^.

The wind tunnel test area and setup were performed as described by van Tol *et al*. (2021).^23^ The light condition in the wind tunnel (ceiling illumination) was adapted to a setting for dusk (Fig. 1) and measured as a reflection of light on a spectralon plate.^23^ The light spectrum was composed of LEDs in the visible range of 400–750 nm (LED‐strip – Full‐color RGB + Warm White – 24 V High Power Protected 5050, LuxaLight, NL)^23^ and a component in the UV‐A range of 360 –390 nm (peak at 365 nm)(strip of UV LED Engin, LZ4‐04UV00, Osram Sylvania Inc., USA). Furthermore, the humidity (70%), temperature (24 °C), and wind speed (2 cm s^–1^) of the tunnel were conditioned based on the optimal data for flight of western flower thrips.^23^

#### 2.2.2 LED wavelength sources

An LED lamp was developed to provide a visual cue for *L. rugulipennis* in the wind tunnel. The lamp consisted of a 3D‐printed black cone (18.5 cm diameter with a diffusing glass plate in the front (11.5 cm diameter) and the inside was covered with aluminum foil (Fig. S1). For each trial, a layer of sticky glue P300I35 (Intercol, Industrial adhesives, NL) was applied on a 100 μm transparent polypropylene sheet (Staples Solutions, NL) and placed on the exterior of the glass plate (Edmund Optics Ltd., York, UK) (Fig. S1). The LED source was placed on the back of the cone, shining through the glass plate, and sticky sheet. The lamp was connected to a holder shining downward at an angle of 45° to the floor of the wind tunnel. The center of the lamp was fixed at 60 cm from the floor, and the insect release platform was placed 70 cm from the base of the lamp and at a height of 8 cm. The selected wavelengths were 365, 385, 405, 420, 470, 490, 530, 590, 650, and 720 nm. Although there are several definitions of the wavelength ranges of colors, the color categories as defined by CIE (https://cie.co.at/publications/cie-collection-photobiology-photochemistry-1999) was adopted for this study. For example, the definition of insect visible UV‐A range is between 315 and 400 nm, and different insects have different optimal UV‐A visibility and response. Therefore, 365 nm was selected as the lowest UV‐A visible wavelength in this study because of safety reasons, particularly, where human exposure is involved. Generally, the wavelengths were selected based on LED color availability and insect visibility as determined by Briscoe and Chittka.^24^ The brightness levels tested were 4.48 and 5.86 μmol photons per steradian m^−2^ s^−1^. The emission spectra of the LEDs and the brightness of the light sources were measured inside the wind tunnel using a broadband spectroradiometer Specbos 1211UV (JETI Technische Instrumente GmbH, Germany).

#### 2.2.3 Wind tunnel experiments

Wind tunnel experiments were performed between May and November 2020, partially overlapping with the dates of the greenhouse experiment, and male and female bugs were tested in response to 10 different wavelengths at two levels of brightness. The light schedule in the climate cabin was similar to the light timing in the experiments (L:D = 16:8 h, light from midnight until 4 pm). After nymphs were transformed into adults, 20 male or female bugs were kept in a small release container without bean pods for 24 h. The release container consisted of a clear plastic box (diameter 8 cm, height 6 cm) covered by a Parafilm (Bemis, PM‐996, USA) layer. At 5 pm, the release container with bugs was placed in the wind tunnel on the release platform, and the bugs were allowed to adapt to the dusk light conditions in the wind tunnel for 1 h after which the parafilm cover was removed to allow the bugs fly in the tunnel. A light source with a chosen wavelength and intensity was activated from the moment the container was placed in the wind tunnel. After the release of the bugs, the experiment ran for 14–15 h until the next morning. The number of bugs on the sticky sheet in front of the light source and the number of bugs that did not leave the release container were recorded.

### 2.3 Greenhouse experiments ‐ visual tests

#### 2.3.1 Water trap design

**Figure 2:**
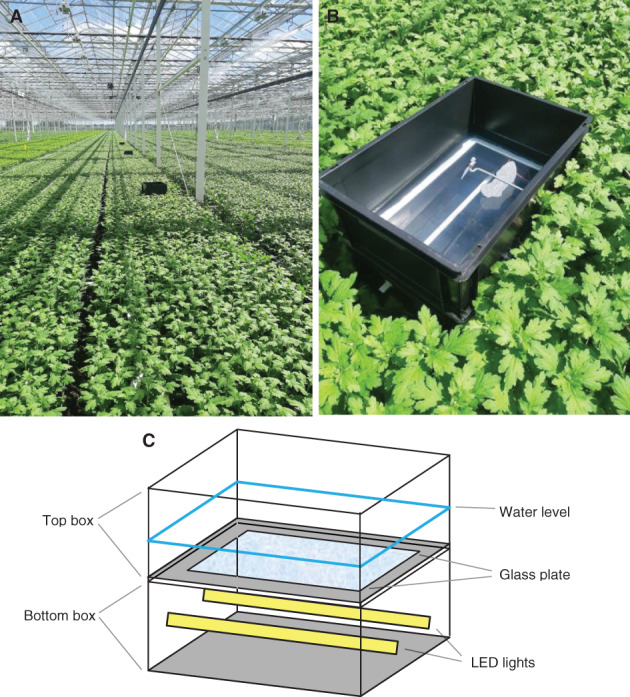
LED water trap in chrysanthemum greenhouse (A) multiple traps, (B) single detailed trap, and (C) schematic details of trap.

**Figure 3:**
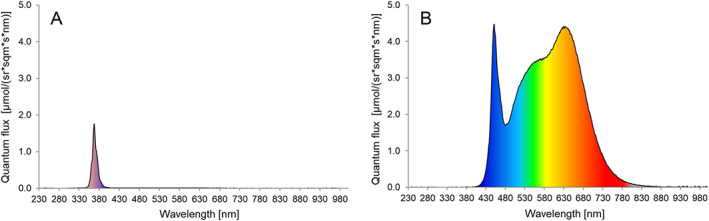
Light intensity (quantum flux = μmol photons per steradian m^−2^ s^−^1 nm^−1^) of LED water trap in chrysanthemum greenhouse for (A) UV‐A (365 nm) or (B) white light (400–780 nm) water trap. Quantum flux UV‐A (343–374 nm) = 19.51, White light (400–780 nm) = 916.10.

Details of the design of the water trap are shown in Fig. 2(C). The trap consisted of two black polypropylene boxes (Manutan, Model RC406, 40 L, length 600, width 400, and height 220 mm) placed on top of each other. The lower box contained two aluminum bars (0.5 m each) on which white LED light strips (LuxaLight Long Life LED‐strip Neutral White (4200–4400 K), https://www.luxalight.eu/en/products/led-strip/luxalight-long-life-led-strip-neutral-white-4300k-protected-24-volt-140-leds) were mounted. The bars were stuck through holes in the lower box, providing a platform for the LED strips and partially sticking outside of the box (5 cm) for cooling purposes of the LED. From the top box, most of the bottom was removed and replaced by a glass plate (armored glass 5 mm thick, length 535 mm, width 335 mm) glued to the bottom edges with silicon. The top box was placed on top of the lower box and half filled with water (110 mm height box, 26.4 L). Tween20 (0.2 mL L^–1^) to reduce the surface tension of the water to trap insects. The wavelengths and intensity of light are shown in Fig. 3.

#### 2.3.2 Greenhouse test

Five LED water traps were placed in two large (5–8 ha) chrysanthemum greenhouses (Made and Zuilichem, the Netherlands) (Fig. 2). A water trap without light and a green Unitrap with pheromone were placed in the greenhouse as negative and positive controls, respectively. The treatments were a water trap with 365 nm LED light and a water trap with LuxaLight Long Life LED‐strip Neutral White light broad spectrum LED, and the traps were placed more than 20 m apart. The trials started on 21 July 2020 and ended on 17 September 2020. The trap light was turned on in the afternoon (approximately 5 pm local time) and turned off at 4 am local time to ensure that the complete dusk flight time was covered. The bugs were collected twice a week from the traps, counted, and then identified.^25^ The water was refreshed and glass plate cleaned at each collection time to avoid reduction of light intensity. The glass plate and box were cleaned solely with paper towels.

### 2.4 GC–MS and GC‐EAD of *L. rugulipennis* extract

Male and female body extracts were collected from the reared bugs by dipping whole bugs in hexane. We collected an extract in 2 mL of hexane from 56 females and 33 males. The bugs were left for 2 h in the extract, after which we removed the extract and condensed it under argon gas to approximately 100 μL. Of the male and female extracts, 1 μL was injected into the GC–MS and profiled (method described in Section 2.5, see supplementary Table S1 for list of identified compounds) and EAD activity for both male and female antennae was determined.^26^

### 2.5 Headspace collection plants with bugs, GC‐EAD and GC–MS analysis

The plants used for odor trapping were lucerne (*Medicago sativa* L.) and chamomile (*Matricaria chamomilla* L.). Plants were grown from seeds in pots and were still in the vegetative stage at the sampling dates. All headspace collections and tests were performed using reared bugs. Plants with 15 male or female *L. rugulipennis* were placed in 2.5 L glass jars in a climate chamber at 24 °C (16 h/8 h light/dark photoperiod). A total of eight equivalent headspace collection jars were setup at the same time in the chamber. Air was purified by passage through an activated charcoal filter and drawn at 0.2 L min^−1^ through a jar. Volatiles were entrained for 48–73 h using Gerstel thermodesorption tubes filled with 80 mg Tenax TA 20/35 mesh (Grace‐Alltech), which were replaced every 12 h. The tubes were cleaned by rinsing with 10 mL hexane and then flushing for 1 h with purified nitrogen (20 mL min^–1^) at 280 °C, and each treatment was repeated twice. A total of two replicate jars for each plant alone and plant with male or female bug volatiles were collected at the same time (in May and September) with the same batch of *L. rugulipennis*: lucerne (total 142 h headspace collection), common groundsel (139 h headspace collection), and chamomile (142 h headspace collection). Additionally, one empty jar was placed for the collection of volatiles at each sampling period (control; 142 h headspace collection). Thereafter, the volatiles were washed off (pooled extract) from the tubes using 5 mL of hexane per Tenax tube, and the total was condensed to approximately 100 μL of concentrated volatiles in hexane. This was performed for all the treatments.

Extracts were analyzed on a Hewlett‐Packard 6890 gas chromatograph, equipped with a split/splitless injector, a Hewlett‐Packard 5973 mass selective detector (70 eV), and Alltech AT‐5 column (30 m × 0.25 mm × 0.5 μm) run in constant flow mode (1.3 mL min^–1^ helium). Injections were performed in splitless mode (1 μL). The oven temperature program was as follows: 1 min at 50 °C followed by heating at 15 °C min^–1^ until 300 °C, and the temperature was maintained at 300 °C for 15 min. When the tentatively identified compound showed similar Kovat indices^27^ with the synthetic reference compound on our chromatographic system, it was considered to be a positive identification. We compared all treatments and tentatively identified compounds that were not found in the controls and were increased or unique when compared to plant odor only (Supplementary Table S1 and Fig. 1). The different headspace collections were tested on the EAD response of male and female antennae using the method and equipment described by van Tol *et al*.^26^ GC‐EAD measurements were performed using an Interscience Trace GC‐2000 equipped with a cold on‐column injector. Purified humidified air was maintained over the antenna at a flow rate of 80 cm s^–1^. The sample was equally split between a flame ionization detector and an EAG detector. Antennae were separated from the weevil heads and mounted between two glass electrodes filled with a Ringer's solution (6.4 mM KCl, 12 mM MgCl20.6H2O, 9.6 mM KOH, 12 mM NaCl, 20 mM KH2PO4, 1 mM CaCl2, and 354 mM glucose in deionized water). Antennal preparation and EAG recording were performed according to the procedure described by Visser and Piron^28^ and van Tol *et al*.^29^ The EAG recorder plus peripheral equipment was manufactured by Syntech Laboratories.

### 2.6 Y‐tube olfactometer experiments

#### 2.6.1 Plant and pheromone attraction

The attraction of male and female *L. rugulipennis* to green bean (*Phaseolus vulgaris*), a sex pheromone product (Agralan, UK), and a combination of bean and pheromone was examined using a Y‐tube olfactometer. The pheromone product contained hexyl butyrate, (*E*)‐2‐hexenyl butyrate, and (*E*)‐4‐oxo‐2‐hexenal. Bugs from a laboratory culture or wild bugs (collected from chamomile and used in tests within 1 month after collection) were used for the experiment. An approximate equivalent leaf mass was cut from each plant and kept wet by placing the base of the leaves in wet cotton covered with aluminum foil. Wet cotton covered in aluminum foil was used as the negative control (blank). After wrapping, they were transferred to a wash bottle (500 mL) with screw caps and Teflon tubes connected to the Y‐tube. The vertically placed Y‐tube olfactometer consisted of a glass Y‐tube (base tube 13 cm long; Y‐arms 12.5 cm long; internal tube diameter 3.5 cm) with a 75° inside angle. A clear plexiglass tube (diameter 3.6 cm; 7 cm long) with gauze on one side and a rubber lid with opening on the other side containing one male or female *L. rugulipennis* was connected to the base tube, which allowed the bug to freely enter the Y‐tube through the opening in the lid. Airflow was purified by passage through an activated charcoal filter wash bottle and a bottle containing water to humidify the air entering the Y‐tube arms. The airflow was set to 5.2 cm s^–1^ (13.2 mL s^–1^). The olfactometer was placed in a black box with a halogen lamp (12 DC, 10 V) in the top center to illuminate the Y‐junction. The light intensity was set to 0.3 × 10^−3^ W m^–2^. Each test lasted 5 min, after which the result was noted, and the bug was removed from the Y‐tube. A gauze in each arm of the Y‐tube prevented the entry of the bugs into the connecting tubes between the wash bottles and the Y‐tube. When the bug had entered one of the arms and passed a line (1 cm from the gauze), the experiment ended. When the bugs did not choose or pass the line in the arms within 5 min, the experiment ended, and a non‐response result was noted. After five bugs were tested, the Y‐tube was removed, washed with hexane, and turned 180° to compensate for possible preference of the Y‐tube arm. Each treatment consisted of a plant with or without pheromone (Agralan, UK) tested against a control (plant or soil only) (Supplementary Table S2). A total of 14–43 bugs (numbers per treatment, see Supplementary Table S2) were tested for each treatment. The number varied according to the availability of the bugs.

#### 2.6.2 Pure compound attraction

Furthermore, the response of the bugs to compounds selected from plant headspace analysis, bug extract analysis, and some compounds referred to as attractive to *Lygus* sp. in publications (Supplementary Table S1) were examined using Y‐tube experiments as described above (Table S5, seven compounds tested, of which two also combined with the sex pheromone). All pure compounds were obtained from Sigma‐Aldrich. The odor compounds were tested either as a release from rubber septa (Pherobank, Wijk bij Duurstede, the Netherlands) or as a pure compound that evaporates via passive penetration through the vial's LDPE container wall (Kartell Labware, Noviglio, Italy). For the rubber septa, we diluted the compound to 1:10 with dichloromethane, and further dilutions with dichloromethane were prepared from the initial dilute. For each dilution, 200 μL was applied to each rubber septum, and the solvent was allowed to evaporate from these septa for 17 h before storage at −20 °C until use in the olfactometer. For solid compounds, the mg/septum was weighed (depending on the density of the compound) and dissolved in dichloromethane, resulting in an identical amount of pure compound per septum as that of the liquid compounds. Different amounts of pure compounds were added to each vial (Kartell), leading to different release rates. The actual amounts of the compounds are listed in Supplementary Table S1. The actual release rates of the compounds were not examined in the present study, thus, information on the actual release rate response was not provided. However, we provided information on the response to dilutions with unknown amounts per mL air of the compounds released in the Y‐tube bioassays.

### 2.7 Statistical analyses

#### 2.7.1 Visual experiments

All data analysis was performed on RStudio with R version 3.4.0.^30^ Raw data were read and organized using the package *tidyverse*.^31^ Responses and predictor variables were checked for collinearity and correlation using the *GGally* package.^32^ A variance inflation factor was computed for the predictors using the *car* package.^33^ Interactions between variables were examined using exploratory graphs created by the *ggplot2* package.^34^ Error bars in all figures represent the standard error of the mean calculated over the entire sample size. Wavelength choice data were analyzed using beta regression with the *betareg*.^35^ Proportion data, and the proportion of bugs attracted was transformed to fit an open interval between 0 and 1.^36^ Data from greenhouse trap experiments, that is, the total number of captured bugs, were analyzed using linear regression. Pairwise comparisons were performed using Tukey's honest significant difference (HSD) test and Kruskal‐Wallis test using the package *agricolae*.^37^

#### 2.7.2 Olfactory experiments

Y‐tube experiment data were analyzed using generalized linear regression with a binomial distribution and logit link. Assumptions from all regression models, such as normality of data and homogeneity of variance were tested using diagnostic plots and statistical tests.^37^ Regression models and their coefficients were further analyzed using Wald tests and Chi‐squared tests.^38^

## 3 RESULTS

### 3.1 Wind tunnel visual experiments

**Figure 4:**
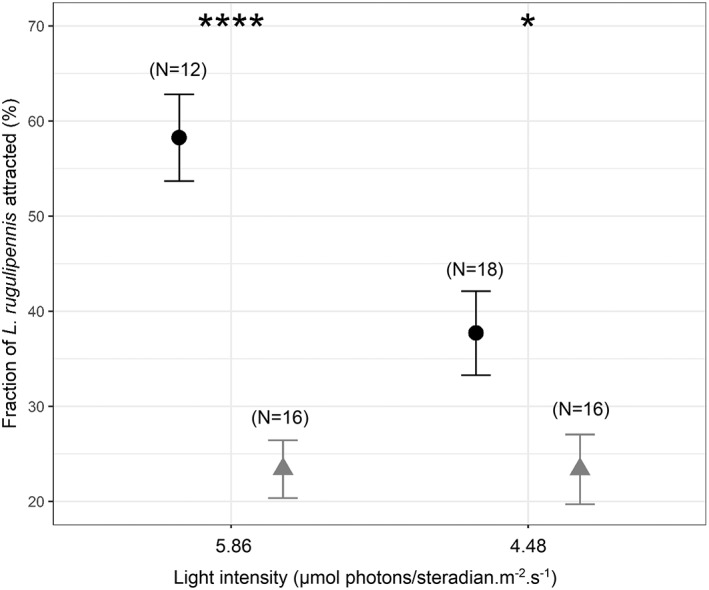
Fraction of total male and female *Lygus rugulipennis* that responded to different wavelength LED light sources in the wind tunnel at two light intensities Black circles represents wavelengths in the range of 365–420 nm (group A, supplementary Table S3: 365, 385, 405, 420 nm) and grey triangles represents wavelengths in the range of 470–720 nm (group B, supplementary Table S3: 470, 490, 530, 590, 650, 720 nm). Statistical differences between the light intensity groups are indicated as ****P < 0.001 and *P < 0.05. Error bars represent the standard error of the mean.

The light setting of the ceiling illumination effectively evoked the flying behavior of the bugs in the wind tunnel. The attraction of Lygus bugs to different light sources (Fig. 4) was significantly affected by the wavelength spectrum (*P* < 0.001, Chi Square = 33.16) and light intensity (*P* < 0.01, Chi Square = 3.93, Beta regression) (Table S3). There was no significance difference (*P* > 0.05) in the response of male and female *L. rugulipennis* to the different light sources. The visual response was largely restricted to wavelengths in the UV‐A/violet range, within which no specific wavelength was superior, according to Tukey's honest significance test. Other wavelengths in the visual range 470–720 nm elicited a much lower response from the bugs. Among the two intensities examined, the bugs were more attracted the higher intensity within the wavelength range of 365–420 nm. However, light intensity did not have a significant impact on attraction within the visual range of 470–720 nm.

### 3.2 Greenhouse experiments – visual tests

**Figure 5:**
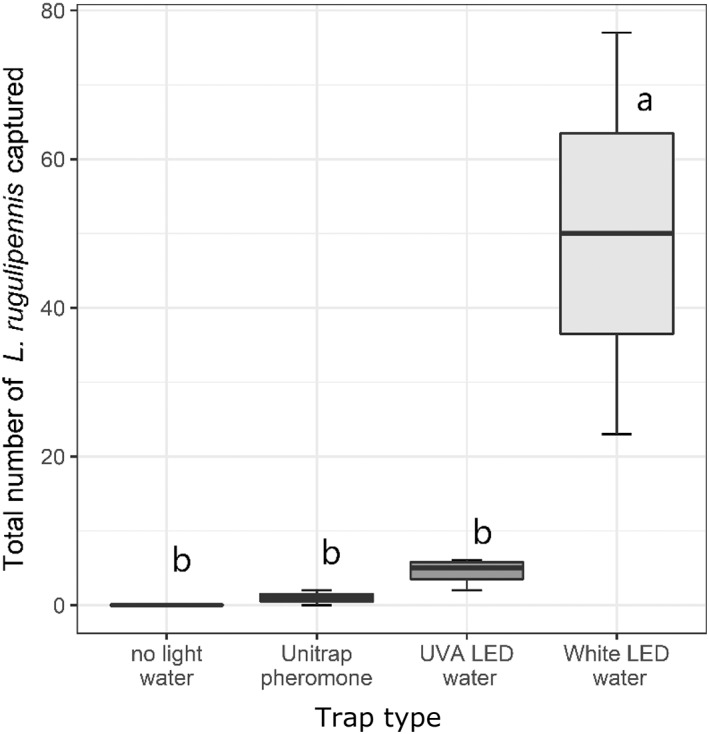
Total number of *Lygus rugulipennis* caught by water traps with no LED light (N = 16), green cross‐vane Unitrap with the Lygus sex pheromone (N = 12), water traps with white LED light (N = 15) or UV‐A LED light (N = 24) in two different chrysanthemum greenhouses during 8 weeks of bug migration flight. The letters indicate statistical differences between the attractiveness of the traps, according to Tukey's HSD test.

The results of the greenhouse experiments showed that there were significant differences in the total number of bugs captured by the different types of traps (*P* = 0.001, Chi Square = 15.98) and during specific weeks (*P* < 0.01, Chi Square = 20.95) (see Supplementary Table S4). In both greenhouses, the bugs were more attracted to the white LED light than the UV‐A or pheromone traps (Fig. 5). Although a few (<1.5%) *L. pratensis* (one specimen) and *L. gemellatus* (three specimens) were captured, the majority (98.5%) of the captured bugs were *L. rugulipennis*. In the two greenhouses, 72 male and 48 female bugs were caught by the LED water traps, whereas only two males were caught by the pheromone trap. The majority of bugs were captured in the same weeks in both greenhouses.

### 3.3 Headspace collection plants with bugs, GC‐EAD and GC–MS analysis

Headspace odors were collected from two undamaged plant species (*M. sativa* and *M. chamomilla*) in the presence of male or female *L. rugulipennis*. A comparison of the GC–MS data of headspace from undamaged plants and plants with male or female bugs identified 10 unique compounds, among which five were unique to bugs [4‐oxo‐(*E*)‐hexenal, hexyl acetate, pentyl butyrate, hexyl butyrate, and (*E*)‐2‐hexenyl butyrate] and two were unique to both bug extracts (Supplementary Fig. S2(C)) and plants infested with bugs ((*E*)‐2‐hexenal, 1‐hexanol) (Supplementary Fig. S2 and Table S1). Several identified compounds, including (*E*)‐*ß*‐ocimene, (*E*)‐DMNT, methyl salicylate, (*E*)‐*β*‐caryophyllene, decanal, were increasingly released from plants in the presence of bugs. GC‐EAD of the plant‐bug headspace revealed electroantennogram responses of both male and female antennae. Positive EAD responses were found for (*E*)‐2‐hexenal, 4‐oxo‐(*E*)‐2‐hexenal, hexyl acetate, hexyl butyrate, pentyl butyrate, (*E*)‐2‐hexenyl butyrate, methyl salicylate, and (*E*)‐*β*‐caryophyllene (Supplementary Table S1).

### 3.4 GC–MS and GC‐EAD of *L. rugulipennis* extract

The headspace of plants with male and female bugs and extracts of male and female bugs were identified by GC–MS. The profiles of the headspace are shown in Supplementary Fig. S2. (*E*)‐hexenal, hexanol, 4‐oxo‐(*E*)‐2‐hexenal, and hexyl butyrate were identified in all profiles, among which positive EAD responses were observed for all the compounds except hexanol (Supplementary Table S1). Pentyl butyrate was only detected in the extract of the bugs, with positive EAD response on the antennae of male and female bugs. Furthermore, (*E*)‐2‐hexenyl butyrate was undetected as it partially overlapped with the high hexyl butyrate peak (Supplementary Fig. S2(C)). Hexyl acetate was found only in the headspace of plants with bugs. Further EAD‐positive responses were observed for (*E*)‐2‐hexenyl butyrate, hexyl acetate, methyl salicylate, and (*E*)‐*β*‐caryophyllene (Supplementary Table S1).

### 3.5 Y‐tube olfactometer experiments

#### 3.5.1 Plant and pheromone attraction

**Figure 6:**
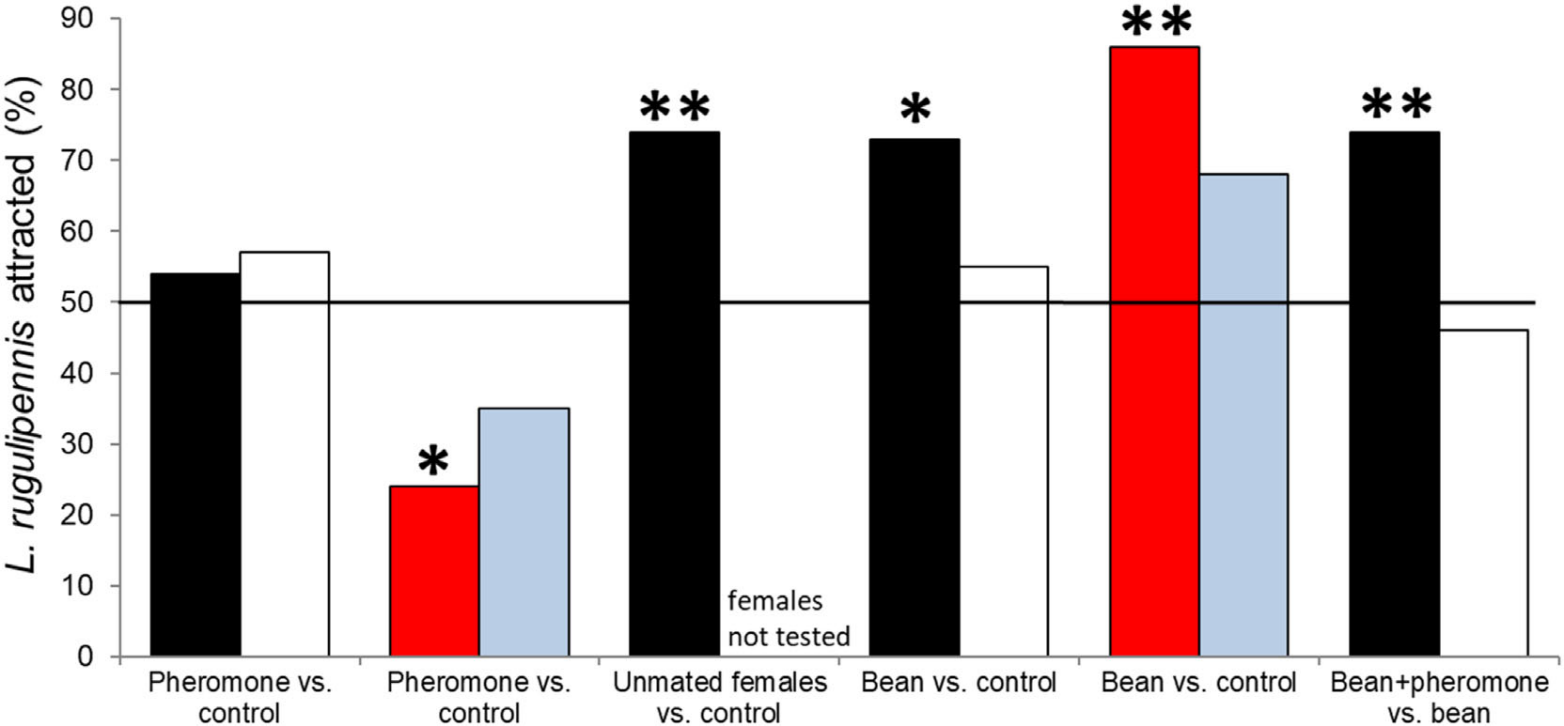
Preference of *Lygus rugulipennis* for a synthetic sex pheromone (Agralan, UK), and unmated females + bean when clean air (control) is the other choice and preference for odor of bean + pheromone when bean odor is the other choice in a Y‐tube olfactometer. Black bars = bean‐reared males, white bars = bean‐reared females, red bars = wild‐collected (Chamomile) males, blue bars = wild‐collected (Chamomile) females. The overall sample sizes ranged from 23–43. Treatment‐specific and sex‐specific sample sizes can be found in supplementary Table S2. Approximately 50% difference in preference indicate statistical significance at *P > 0.05 or **P > 0.01.

The results of the Y‐tube olfactometer are summarized in Supplementary Table S2. The response of male and female bugs to the synthetic pheromone was not significant [*p* (>Chi Square) = 0.63]. However, rearing conditions (bean‐reared or wild‐reared) significantly affected [*p* (>Chi Square) < 0.01] the response of the bugs to the synthetic pheromone. Bean‐reared male bugs exhibited no response to the synthetic pheromone, whereas wild‐reared male bugs exhibited negative response to the pheromone. Regarding bean‐reared bugs, the response of the bugs to combined plant odor and pheromone was significantly affected [*p* (>Chi Square) < 0.05] by sex. Male bugs had a positive response towards bean and pheromone odors, whereas female bugs did not (Fig. 6). Male bugs responded positively to odors from unmated females, which differs from their response to synthetic pheromones.

#### 3.5.2 Pure compound attraction

**Figure 7:**
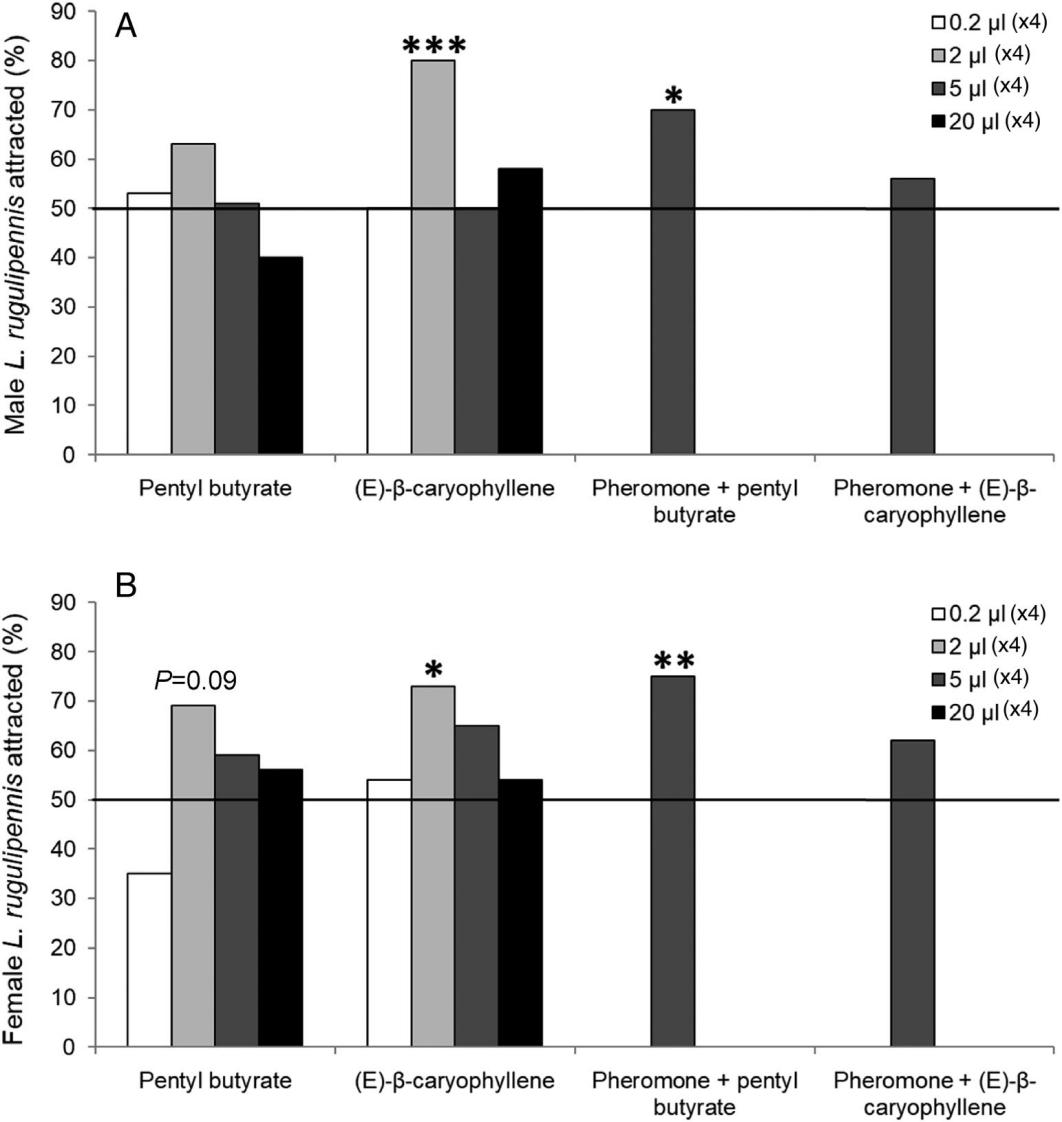
Preference of (A) male and (B) female *Lygus rugulipennis* for synthetic compounds supplied in different amounts in rubber septa in the presence or absence of synthetic sex pheromone (Agralan, UK) in a Y‐tube olfactometer. The overall sample sizes ranged from 22–32 for the synthetic compounds supplied in Kartell tubes, and it ranged from 26–32 for the synthetic liquid compounds supplied in the rubber septa. Treatment‐specific, dose‐specific, and sex‐specific sample size can be found in supplementary Table S5. Approximately 50% difference in preference indicate statistical significance at *P > 0.05, **P > 0.01 or ***P > 0.001.

The results of the response of the bugs to different quantities of the compounds is summarized in Supplementary Tables S5(a) and (b). The amount of liquid compounds applied per rubber septum and the amount applied in mg in Kartell's is shown in Supplementary Tables S5(a) and (b), respectively. Positive attraction was observed towards two of the tested compounds, with and without synthetic pheromones (Fig. 7). There was a near dose‐dependent attraction to pentyl butyrate [*p*(>Chi Square) = 0.09) and a dose‐dependent attraction to (*E*)‐*ß*‐caryophyllene [*p*(>Chi Square) < 0.05], according to a generalized linear regression. However, there was no significant difference in the response of male and female bugs to pentyl butyrate [*p*(>Chi Square) = 0.65] and (*E*)‐*β‐*caryophyllene [*p*(>Chi Square) = 0.85]. We also measured the release rate (in mg weight loss) from the septa of these attractive compounds as this is an important value for dose‐ air concentration response of the bugs. The release rate of 2 μL of (*E*)‐*ß*‐caryophyllene or pentyl butyrate/septum was between 0.11 and 0.27 mg per 24 h for (*E*)‐*ß*‐caryophyllene and between 0.11 and 0.32 mg per 24 h for pentyl butyrate. Among the amounts examined, the bugs were attracted to only 2 μL. Furthermore, females were attracted to both compounds, whereas males were attracted to only (*E*)‐*ß*‐caryophyllene. The release rates of the other amounts applied per septum were not measured, and no data on release rates were provided.

Furthermore, the response of the bugs to the attractive amounts (2 μL) of both compounds in combination with the synthetic pheromone (5 μL of each kairomone + separate sex pheromone pipette dispenser) was examined. Responses to pentyl butyrate and pheromone were positive for both females and males, whereas combining the pheromone with (*E*)‐*ß*‐caryophyllene appeared to be less attractive. Among the other tested compounds (Supplementary Table S5), the bugs exhibited a dose‐dependent response [*p*(>Chi Square) < 0.05] to only decanal. The bugs were only attracted to the lowest quantity of decanal (0.2 μL). Additionally, male bugs were only attracted to 50 mg per Kartell vial of 1,4‐dimethoxybenzene. However, the actual release rates of the compounds were not examined in the present study.

## 4 DISCUSSION AND CONCLUSIONS

Over the years, it is believed that olfactory attraction is more effective than visual attraction in trapping *L. rugulipennis*. Compared with pheromone traps, which have been reported to capture only male bugs in low numbers,^11^ the water trap with white LED light, however, caught approximately 20–30 times more bugs of both sexes in the present study. Although we could not ascertain whether the distance between the traps (>20 m) was large enough to prevent interaction between the treatments, the significant difference between the number of bugs caught by UV‐A and white LED traps indicated that trap distance was large enough to distinguish the color preference of the bugs. Several kairomonal compounds and potential minor pheromonal compounds appear attractive to male and female bugs in Y‐tube experiments but not in a greenhouse (van Tol, unpublished).

The finding of the present study showed that the bugs were more responsive to white light than to UV‐A light. Moreover, the wind tunnel experiments indicated a strong response to the UV‐A/violet range (365–420 nm) and weak to no response to the other wavelengths examined. However, white light, which is a combination of all wavelengths has not been tested in a wind tunnel and may be more attractive than UV‐A to the bugs. Since several day and night active insect species have been reported to be attracted to UV‐A,^22^, ^39^, ^40^, ^41^ we speculated a similar response for *L. rugulipennis* in greenhouse and wind tunnel experiments. Although *Nesidiocorus tenuis* Reuter has been found to be more attracted to UV lights of 385 nm wavelength than broad white light in a laboratory experiment,^22^ studies are yet to determine whether members of the Miridae family are attracted to UV lights in greenhouses. Šedivý and Honěk^18^ reported that *L. rugulipennis* was attracted to white light containing 25% UV‐A light in the field. However, the combination of UV‐A with white light does not necessarily influence bug attraction significantly, because the white light water trap without UV‐A captured more bugs than UV‐A only (365 nm). Additionally, light intensity may play a considerable role in attracting Lygus bugs. In the present study, the white light trap was 47 times brighter than the UV trap in the greenhouse test. However, the wind tunnel experiments indicated that the bugs had a higher preference for UV/violet, but not for any of the other wavelengths examined. The water trap with UV‐A light attracted some male and female bugs (1 to 4 per trap per week; 42% males and 58% females), but was not significantly different from the number of males caught with the pheromone trap (0 to 1 per trap per week; 100% males). Comparing our results with the preference for sunlight, reflecting colored traps seem less appropriate as the insects become active during dusk.^11^, ^18^, ^21^ Colored traps rely on sunlight reflection which is absent or limited at dusk conditions. Therefore, brightness may likely explain the preference for certain colors by dusk flying insects, even though many night insects appear to have color vision.^42^

The practicability of the sex pheromone trap *versus* the water trap with LED light depends on several factors, including cost. The current water trap is approximately 4–5 times more expensive than the pheromone trap. A sex pheromone product (with two replacement pheromone dispensers) costs approximately 75 Euro in the Netherlands and is effective for approximately 4 months, whereas water trap with LED light, LED trafo, and 30 m cable is currently produced at 340 Euro per trap. Additionally, the pheromone dispenser needs to be refreshed every 6–8 weeks, whereas the water trap does not incur additional costs over the years, except for electricity costs. Moreover, LED lights use less electricity and are required only during the night. Furthermore, the findings of the present study showed that both male and female bugs were attracted to LED trap, whereas only a few males were caught by the pheromone trap. Although some night flying moths and flies were caught by water traps, the number caught was small (no counting performed). Another limitation of light traps is light pollution at night, particularly in Chrysanthemum greenhouses. The traps have to be used for several weeks per year (two migration flights per year), and as such, it seems less problematic than the general light pollution of greenhouses. Although water traps are currently useful for improved monitoring, it is unclear if they are suitable tools for the mass trapping of insect pests. An increase in bug invasion into greenhouses (luring) by the light of the traps is possible, but seems less likely. The current pheromone trap rarely predicts these flights correctly, and the light trap does not seem to increase catches in the pheromone trap. Over the years, there have been on‐going discussions on the efficacy of olfactory and visual stimuli to attract pests in greenhouses. For example, the response of western flower thrips to attractants has been examined and no additional attraction from outside into the greenhouse has been observed.^43^ Prevention of pest invasion by netting is usually too expensive (installation and maintaining/cleaning costs) for most growers. All trap types catch only migrating adults; therefore, combining traps that capture migrating adults with a ‘push‐pull’ strategy^8^ could be effective in preventing or reducing pest damage.

Furthermore, some compounds that were attractive to both male and female *L. rugulipennis* were identified in the semiochemical research. The bugs were attracted to a combination of commercially available synthetic sex pheromones and plant odors, indicating an interaction between commercially available synthetic sex pheromones and plant odors. However, the pheromone alone (bean‐reared *Lygus*) was not attractive to the bugs, and strongly repelled wild‐collected males. Moreover, more males were attracted to beans with pheromones than to beans alone. Additionally, more males (74%) were attracted to pheromones released by unmated females than to the synthetic pheromone released in the Y‐tube olfactometer. Furthermore, studies have shown that several behavioral characteristics of females (attraction, repellence, aggregation, and spacing) are determined by the ratios and amounts of the pheromone components released.^12^, ^13^, ^44^, ^45^, ^46^, ^47^ However, studies are yet to verify whether some other minor plant compounds released by the bugs, such as (*E*)‐2‐hexenal and 1‐hexanol, play a role in attraction. Some studies have reported that (*E*)‐2‐hexenal and 1‐hexanol are found in the metathoracic glands, and they may play a role in pheromone attraction.^44^, ^48^ These components are currently absent in the synthetic pheromone product.

In the present study, compounds identified in the headspace of *M. sativa* and *Matricaria chamomilla* in the presence and absence of male or female *L. rugulipennis* are presented in Supplementary Table S1. The important sexually attractive compound 4‐oxo‐(*E*)‐hexenal was only released by females in the headspace of both plant species and was found in nearly equal amounts in the extract of female and male bugs (Supplementary Fig. S2(C)). Innocenzi *et al*.^10^ showed that 4‐oxo‐(*E*)‐hexenal in combination with hexyl butyrate plays a key role in male attraction, with similar results observed in other *Lygus* species.^44^, ^48^ Furthermore, other compounds, including hexyl acetate, *ß*‐ocimene, methyl salicylate, (*E*)‐4,8‐dimethyl‐1,3,7‐nonatriene, and *ß*‐caryophyllene, were identified in the headspace of the plants after bug damage. Most of these compounds do not seem to play a role in attraction; however, they may trigger attraction or repellence in combination with pheromone odor.^49^ One EAD‐active compound (pentyl butyrate) was identified in the bug extract but not in the headspace of the bugs. Therefore, the response of bugs to this compound was examined using the Y‐tube method.

From all listed compounds identified by GC–MS, the response of the bugs to pentyl butyrate (*E*)‐2‐hexenal, (*E*)‐*ß*‐caryophyllene, and decanal was examined using the Y‐tube olfactometer. Additionally, the response of the bugs to some compounds, including phenylacetaldehyde, 2‐phenyl ethanol, and 1,4‐dimethoxybenzene, which have been previously listed as attractive or potentially attractive to Hemipterans was examined.^8^, ^11^, ^14^, ^15^, ^16^, ^50^ Among the compounds examined, the bugs exhibited a dose‐dependent response to pentyl butyrate and (*E*)‐*ß*‐caryophyllene in the Y‐tube (Fig. 7). Pentyl butyrate is an unknown compound found in the extracts of *L. rugulipennis*. Tasin *et al*.^51^ reported that grapevine moth was attracted to (*E*)‐*ß*‐caryophyllene. Furthermore, a comparison of the response of the bugs to the synthetic pheromone alone and a combination of the pheromone and the compounds showed that both male and female bugs were attracted to a combination of the synthetic pheromone and pentyl butyrate, in contrast to the negative response of the bugs to the synthetic pheromone alone (Supplementary Table S5). However, the bugs were not attracted to a combination of (*E*)‐*ß*‐caryophyllene and the synthetic pheromone, indicating a negative interaction between (*E*)‐*ß*‐caryophyllene and the pheromone. The reason for this negative interaction requires further investigation.

The visual attraction of bugs to water traps with white LED light could be promising for the monitoring and mass‐trapping of *L. rugulipennis*. Olfactory attraction to novel semiochemicals has so far only been shown in Y‐tube experiments and appears to be concentration‐related. However, determining an effective dose of the attractive compounds at different distances from the release source may be challenging. Further studies are necessary to examine the effectiveness of water trap with LED light for mass‐trapping of *L. rugulipennis* and other pests of the *Lygus* genera.

## Supporting information


**Figure S1**. Set‐up visual wind tunnel experiments. (A) Test arena with ceiling light, LED source and release platform. (B) Detail of LED light source with clear sticky sheet in front of the LED cone.

**Figure S2**. GC–MS profile of headspace of male and female *Lygus rugulipennis* on (A) *Medicago sativa* and (B) *Matricaria chamomilla* after deduction of GC–MS values of headspace of host‐plant without *Lygus rugulipennis*, (C) extract of males and females in hexane. Retention time of (C) is different from (A) and (B) due to different heating program in the GC–MS. Tentatively identified compounds: 1 = (*E*)‐2‐hexenal, 2 = hexanol, 3 = 4‐oxo‐(*E*)‐hexenal, 4 = hexyl acetate, 5 = (*E*)‐*ß*‐ocimene, 6 = pentyl butyrate, 7 = (*E*)‐4,8‐dimethyl‐1,3,7‐nonatriene, 8 = hexyl butyrate, 9 = methyl salicylate, 10 = (*E*)‐*ß*‐caryophyllene.

**Table S1**. GC–MS identified headspace compounds of plants + *Lygus rugulipennis* and *Lygus rugulipennis* extract unique or increased and EAD response for testing in a Y‐tube olfactometer on behavioural response of *Lygus rugulipennis* males and females

**Table S2**. Preference (%) of *Lygus rugulipennis* for plant odors and pheromone in a Y‐tube olfactometer

**Table S3**. Proportional attraction (%) of *Lygus rugulipennis* to different light sources in wind tunnel visual experiments

**Table S4**. Total number of species‐specific *Lygus* spp. bugs captured in greenhouse visual experiments, summarized over different weeks of capture

**Table S5a**. Preference (%) of *Lygus rugulipennis* for synthetic liquid compounds applied on rubber septa in a Y‐tube olfactometer
**Table S5b**. Preference (%) of *L. rugulipennis* for synthetic compounds applied in Kartell's in a Y‐tube olfactometer

## Data Availability

The data that support the findings of this study are available from the corresponding author upon reasonable request.
