# Supplementary material for: Visual attraction of the European tarnished plant bug Lygus rugulipennis (Hemiptera: Miridae) to a water trap with LED light in chrysanthemum greenhouses and olfactory attraction to novel compounds in Y‐tube tests
Source: Pest Manag Sci. 2022 Apr 6;78(6):2523–33. doi: 10.1002/ps.6881 (PMC9323443; doi:10.1002/ps.6881)
Supplement: Supplementary file 1 — Figure S1. Set‐up visual wind tunnel experiments. (A) Test arena with ceiling light, LED source and release platform. (B) Detail of LED light source with clear sticky sheet in front of the LED cone. [file PS-78-2523-s006.docx]

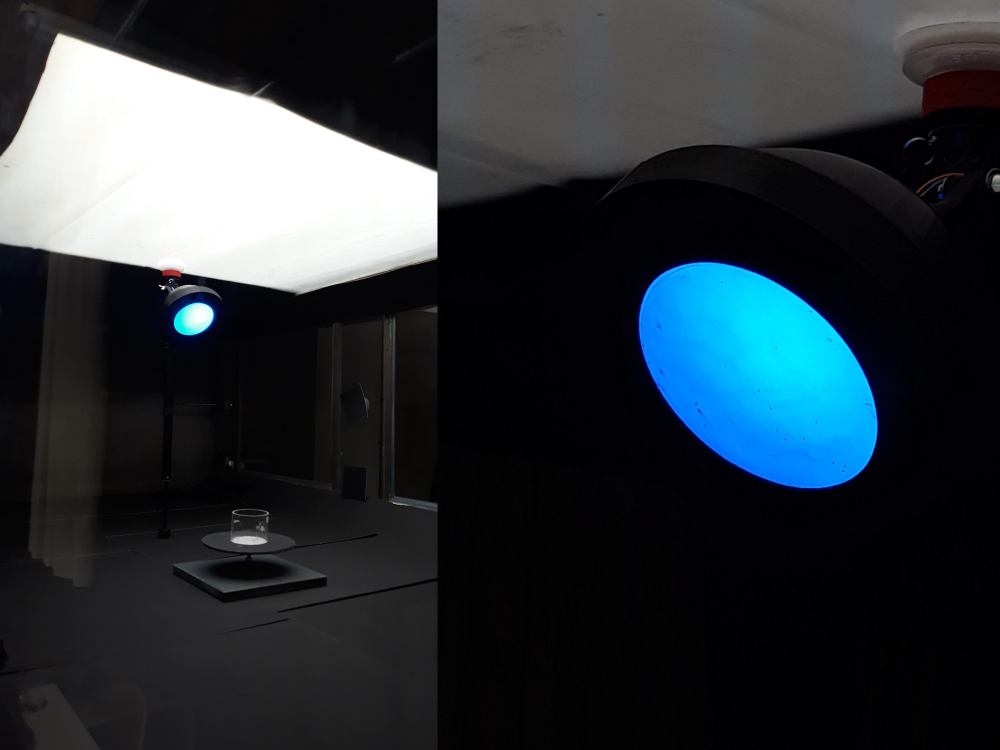


A

B

Figure S1. Set-up visual wind tunnel experiments. (A) test arena with ceiling light, LED source and release platform. (B) detail of LED light source with clear sticky sheet in front of the LED cone.
