# Supplementary material for: Visual attraction of the European tarnished plant bug Lygus rugulipennis (Hemiptera: Miridae) to a water trap with LED light in chrysanthemum greenhouses and olfactory attraction to novel compounds in Y‐tube tests
Source: Pest Manag Sci. 2022 Apr 6;78(6):2523–33. doi: 10.1002/ps.6881 (PMC9323443; doi:10.1002/ps.6881)
Supplement: Supplementary file 2 — Figure S2. GC–MS profile of headspace of male and female Lygus rugulipennis on (A) Medicago sativa and (B) Matricaria chamomilla after deduction of GC–MS values of headspace of host‐plant without Lygus rugulipennis, (C) extract of males and females in hexane. Retention time of (C) is different from (A) and (B) due to different heating program in the GC–MS. Tentatively identified compounds: 1 = (E)‐2‐hexenal, 2 = hexanol, 3 = 4‐oxo‐(E)‐hexenal, 4 = hexyl acetate, 5 = (E)‐ß‐ocimene, 6 = pentyl butyrate, 7 = (E)‐4,8‐dimethyl‐1,3,7‐nonatriene, 8 = hexyl butyrate, 9 = methyl salicylate, 10 = (E)‐ß‐caryophyllene. [file PS-78-2523-s004.docx]

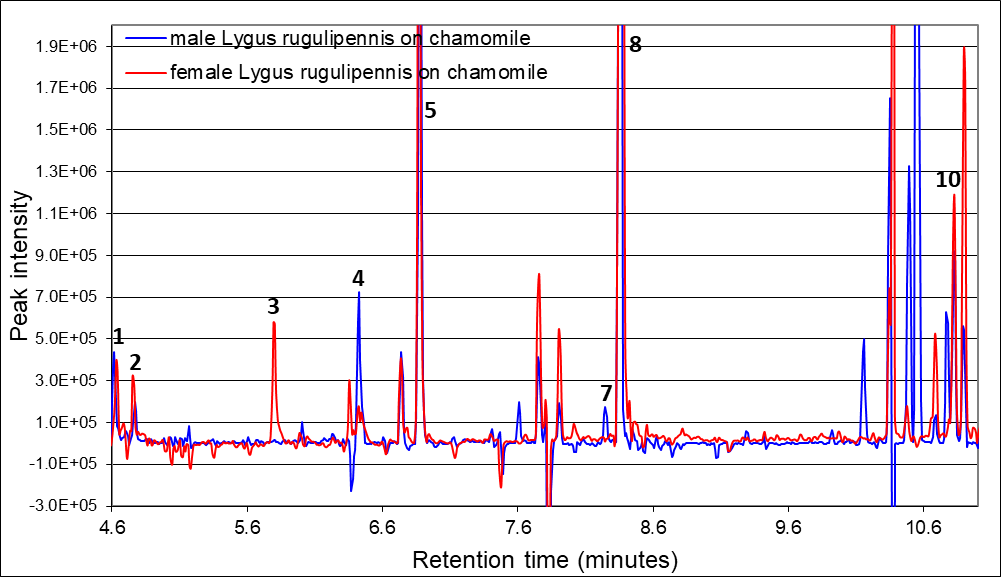

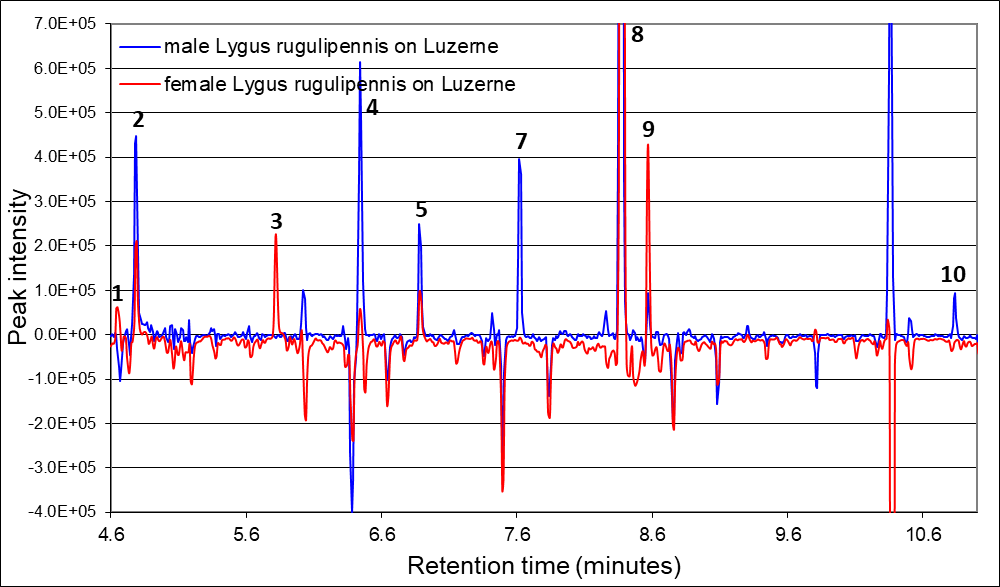


B

A


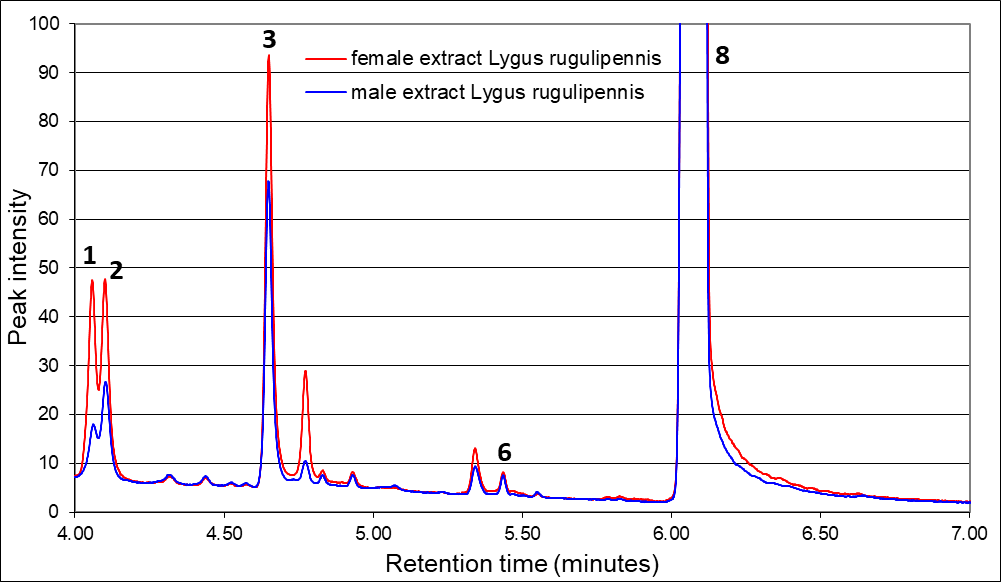


C

Figure S2. GC-MS profile of headspace of male and female *Lygus rugulipennis* on (A) *Medicago sativa* and (B) *Matricaria chamomilla* after deduction of GC-MS values of headspace of host-plant without *Lygus rugulipennis*, (C) extract of males and females in hexane. Retention time of (C) is different from (A) and (B) due to different heating program in the GC-MS. Tentatively identified compounds: 1=(*E*)-2-hexenal, 2=hexanol, 3=4-oxo-(*E*)-hexenal, 4=hexyl acetate, 5=(*E*)-*ß*-ocimene, 6=pentyl butyrate, 7=(*E*)-4,8-dimethyl-1,3,7-nonatriene, 8=hexyl butyrate, 9=methyl salicylate, 10=(*E*)-*ß*-caryophyllene.
