# Supplementary material for: Visual attraction of the European tarnished plant bug Lygus rugulipennis (Hemiptera: Miridae) to a water trap with LED light in chrysanthemum greenhouses and olfactory attraction to novel compounds in Y‐tube tests
Source: Pest Manag Sci. 2022 Apr 6;78(6):2523–33. doi: 10.1002/ps.6881 (PMC9323443; doi:10.1002/ps.6881)
Supplement: Supplementary file 3 — Table S1. GC–MS identified headspace compounds of plants + Lygus rugulipennis and Lygus rugulipennis extract unique or increased and EAD response for testing in a Y‐tube olfactometer on behavioural response of Lygus rugulipennis males and females [file PS-78-2523-s005.docx]

Table S1. GC-MS identified headspace compounds of plants + *Lygus rugulipennis* and *Lygus rugulipennis* extract unique or increased and EAD response for testing in a Y-tube olfactometer on behavioural response of *Lygus rugulipennis* males and females

| Compound | Source^*^ | EAD response^#^ | Y-tube bioassay | Amount  tested^$^ |
| --- | --- | --- | --- | --- |
| (*E*)-2-hexenal | (1), (2), (4) | + | yes | 0.2, 5, 20 µl |
| Hexanol | (1), (2), (4) | - | no |  |
| 4-oxo-(*E*)-2-hexenal | (1), (2), (3), (4) | + | no |  |
| Pentyl butyrate | (4) | + | yes | 0.2, 2, 5, 20 µl |
| Hexyl acetate | (1), (2) | + | no |  |
| Hexyl butyrate | (1), (2), (4) | + | no |  |
| (*E*)-2-hexenyl butyrate | (1), (2), (3) | + | no |  |
| (*E*)-*ß*-ocimene | (1), (2) | - | no |  |
| (*E*)-DMNT | (1), (2), (3) | - | no |  |
| Methyl salicylate | (2) | + | no |  |
| (*E*)-*ß*-caryophyllene | (1), (2), (3) | + | yes | 0.2, 2, 5, 20 µl |
| decanal | (3) | not tested | yes | 0.2, 5, 20 µl |
| 2-phenyl ethanol | literature | not tested | yes | 2, 10, 20 mg |
| 1,4-dimethoxybenzene | literature | not tested | yes | 0.1, 1, 2, 50, 100, 200 mg |
| Phenylacetaldehyde | literature | not tested | yes | 0.2, 2, 5, 20 µl |

* headspace values unique or increased from plants + bugs versus plants alone or from extracted bugs; (1) = *Matricaria chamomilla*, (2) *Medicago sativa*, (3) *Senecio vulgaris*, (4) extract ♂ and ♀ *Lygus rugulipennis*

# No response means no response to the amount as released by plant + bug headspace

$ amount in rubber septum in µl; amount in Kartell in mg
