# Supplementary material for: Visual attraction of the European tarnished plant bug Lygus rugulipennis (Hemiptera: Miridae) to a water trap with LED light in chrysanthemum greenhouses and olfactory attraction to novel compounds in Y‐tube tests
Source: Pest Manag Sci. 2022 Apr 6;78(6):2523–33. doi: 10.1002/ps.6881 (PMC9323443; doi:10.1002/ps.6881)
Supplement: Supplementary file 4 — Table S2. Preference (%) of Lygus rugulipennis for plant odors and pheromone in a Y‐tube olfactometer [file PS-78-2523-s001.docx]

Table S2. Preference (%) of *Lygus rugulipennis* for plant odours and pheromone in a Y-tube olfactometer

| Treatment | Bean-reared bugs | | | | Wild-collected bugs (Chamomile) | | | |
| --- | --- | --- | --- | --- | --- | --- | --- | --- |
|  | N♂ | choice | N♀ | choice | N♂ | choice | N♀ | choice |
| Control | 32 | 27 | 14 | 45 | 24 | 14 | 30 | 32 |
| Bean |  | 73* |  | 55 |  | 86*** |  | 68 |
| Control | 41 | 46 | 34 | 43 | 34 | 76* | 30 | 65 |
| Pheromone |  | 54 |  | 57 |  | 24 |  | 35 |
| Bean | 41 | 26 | 23 | 54 |  |  |  |  |
| Bean+pheromone |  | 74** |  | 46 |  |  |  |  |
| Control | 43 | 26 |  |  |  |  |  |  |
| Unmated females |  | 74** |  |  |  |  |  |  |

preference different from 50% are statistically significant at * P>0.05, ** P>0.01 or *** P>0.001
