# Supplementary material for: Visual attraction of the European tarnished plant bug Lygus rugulipennis (Hemiptera: Miridae) to a water trap with LED light in chrysanthemum greenhouses and olfactory attraction to novel compounds in Y‐tube tests
Source: Pest Manag Sci. 2022 Apr 6;78(6):2523–33. doi: 10.1002/ps.6881 (PMC9323443; doi:10.1002/ps.6881)
Supplement: Supplementary file 5 — Table S3. Proportional attraction (%) of Lygus rugulipennis to different light sources in wind tunnel visual experiments [file PS-78-2523-s007.docx]

Table S3. Proportional attraction (%) of *Lygus rugulipennis* to different light sources in wind tunnel visual experiments.

| **Wavelength group** | **Light intensity (µmol photons/sr.m^-2^.s^-1^)** | **Replicate** | **Total number of responders** | **Sample size (N)** | **Proportion of bugs attracted (%)** | |
| --- | --- | --- | --- | --- | --- | --- |
|  |  |  |  |  | **Mean** | **Standard deviation** |
| A | 5.86 | 1 | 176 | 6 | 63.2 | 0.15 |
|  |  | 2 | 172 | 6 | 53.2 | 0.06 |
| A | 4.48 | 1 | 170 | 8 | 48.9 | 0.09 |
|  |  | 2 | 248 | 6 | 27.5 | 0.1 |
|  |  | 3 | 154 | 2 | 26 | NA |
|  |  | 4 | 124 | 2 | 30.6 | NA |
| B | 5.86 | 1 | 346 | 10 | 19.1 | 0.12 |
|  |  | 2 | 294 | 6 | 30.5 | 0.06 |
| B | 4.48 | 1 | 372 | 12 | 25.1 | 0.12 |
|  |  | 2 | 208 | 4 | 19.2 | 0.02 |

Wavelength group A refers to wavelengths in the range of 365 to 420 nm and wavelength group B refers to the range of 470 to 720 nm.
