# Supplementary material for: Visual attraction of the European tarnished plant bug Lygus rugulipennis (Hemiptera: Miridae) to a water trap with LED light in chrysanthemum greenhouses and olfactory attraction to novel compounds in Y‐tube tests
Source: Pest Manag Sci. 2022 Apr 6;78(6):2523–33. doi: 10.1002/ps.6881 (PMC9323443; doi:10.1002/ps.6881)
Supplement: Supplementary file 6 — Table S4. Total number of species‐specific Lygus spp. bugs captured in greenhouse visual experiments, summarized over different weeks of capture [file PS-78-2523-s002.docx]

Table S4. Total number of species-specific *Lygus* spp. bugs captured in greenhouse visual experiments, summarized over different weeks of capture.

| **Green**  **house** | **Type of trap** | **Sample size (N)** | ***Lygus rugulipennis*** | ***Lygus gemellatus*** | ***Lygus pratensis*** |
| --- | --- | --- | --- | --- | --- |
| Krelingen | no-light-water | 8 | 0 | 0 | 0 |
| Middelburg | no-light-water | 8 | 0 | 0 | 0 |
| Krelingen | Unitrap-pheromone | 6 | 2 | 0 | 0 |
| Middelburg | Unitrap-pheromone | 6 | 0 | 0 | 0 |
| Krelingen | UVA-LED-water | 4 | 5 | 0 | 0 |
| Krelingen | UVA-LED-water | 4 | 6 | 0 | 0 |
| Krelingen | UVA-LED-water | 4 | 6 | 0 | 0 |
| Krelingen | UVA-LED-water | 4 | 2 | 0 | 0 |
| Middelburg | UVA-LED-water | 4 | 5 | 0 | 0 |
| Middelburg | UVA-LED-water | 4 | 3 | 0 | 0 |
| Krelingen | white-LED-water | 8 | 75 | 1 | 1 |
| Middelburg | white-LED-water | 7 | 23 | 0 | 0 |
