# Supplementary material for: Visual attraction of the European tarnished plant bug Lygus rugulipennis (Hemiptera: Miridae) to a water trap with LED light in chrysanthemum greenhouses and olfactory attraction to novel compounds in Y‐tube tests
Source: Pest Manag Sci. 2022 Apr 6;78(6):2523–33. doi: 10.1002/ps.6881 (PMC9323443; doi:10.1002/ps.6881)
Supplement: Supplementary file 7 — Table S5a. Preference (%) of Lygus rugulipennis for synthetic liquid compounds applied on rubber septa in a Y‐tube olfactometer Table S5b. Preference (%) of L. rugulipennis for synthetic compounds applied in Kartell's in a Y‐tube olfactometer [file PS-78-2523-s003.docx]

Table S5a. Preference (%) of *Lygus rugulipennis* for synthetic liquid compounds applied on rubber septa in a Y-tube olfactometer

| Treatment | 0.2 µl | | | | 2 µl | | | | 5 µl | | | | 20 µl | | |  |
| --- | --- | --- | --- | --- | --- | --- | --- | --- | --- | --- | --- | --- | --- | --- | --- | --- |
|  | N♂ | choice | N♀ | choice | N♂ | choice | N♀ | choice | N♂ | choice | N♀ | choice | N♂ | choice | N♀ | choice |
| Control | 32 | 53 | 29 | 42 |  |  |  |  | 31 | 59 | 30 | 57 | 26 | 46 | 30 | 54 |
| (*E*)-2-hexenal |  | 47 |  | 58 |  |  |  |  |  | 41 |  | 43 |  | 54 |  | 46 |
| Control | 30 | 47 | 31 | 65 | 30 | 37 | 31 | 31 | 31 | 49 | 32 | 41 | 32 | 60 | 30 | 44 |
| Pentyl butyrate |  | 53 |  | 35 |  | 63 |  | 69^#^ |  | 51 |  | 59 |  | 40 |  | 56 |
| Control | 26 | 50 | 30 | 46 | 31 | 20 | 32 | 27 | 30 | 50 | 31 | 35 | 31 | 42 | 31 | 46 |
| (*E*)-*ß*-caryophyllene |  | 50 |  | 54 |  | 80*** |  | 73* |  | 50 |  | 65 |  | 58 |  | 54 |
| Control | 31 | 24 | 29 | 45 |  |  |  |  | 29 | 45 | 31 | 67 | 26 | 58 | 31 | 52 |
| Decanal |  | 76** |  | 55 |  |  |  |  |  | 55 |  | 33 |  | 42 |  | 48 |
| Control | 30 | 50 | 29 | 51 | 31 | 54 | 31 | 46 | 29 | 55 | 31 | 65 | 31 | 36 | 27 | 45 |
| Phenylacetaldehyde |  | 50 |  | 49 |  | 46 |  | 54 |  | 45 |  | 35 |  | 64 |  | 55 |
| Control |  |  |  |  | 27 | 30 | 28 | 25 |  |  |  |  |  |  |  |  |
| Pentyl butyrate+pheromone |  |  |  |  |  | 70* |  | 75** |  |  |  |  |  |  |  |  |
| Control |  |  |  |  | 27 | 44 | 29 | 38 |  |  |  |  |  |  |  |  |
| (*E*)-*ß*-caryophyllene+pheromone |  |  |  |  |  | 56 |  | 62 |  |  |  |  |  |  |  |  |

preference different from 50% are statistically significant at * P>0.05, ** P>0.01 or *** P>0.001. Near significant ^#^ P>0.1

Table S5b. Preference (%) of *L. rugulipennis* for synthetic compounds applied in Kartell’s in a Y-tube olfactometer

| Treatment | 2 mg | | | | 10 mg | | | | 20 mg | | | | 50 mg | | |  |
| --- | --- | --- | --- | --- | --- | --- | --- | --- | --- | --- | --- | --- | --- | --- | --- | --- |
|  | N♂ | choice | N♀ | choice | N♂ | choice | N♀ | choice | N♂ | choice | N♀ | choice | N♂ | choice | N♀ | choice |
| Control | 27 | 33 | 28 | 43 | 28 | 57 | 32 | 50 | 29 | 42 | 31 | 52 |  |  |  |  |
| 2-Phenyl ethanol |  | 67 |  | 57 |  | 43 |  | 50 |  | 58 |  | 48 |  |  |  |  |
| Control | 22 | 37 | 21 | 47 |  |  |  |  |  |  |  |  | 29 | 48 | 32 | 31 |
| 1,4-Dimethoxybenzene^#^ |  | 63 |  | 53 |  |  |  |  |  |  |  |  |  | 52 |  | 69* |

# amounts lower than 2 mg (0.1 and 1 mg) and higher than 50 mg (100 and 200 mg) were tested (not shown) but no significant attraction was found
